# Lung Cancer and Cardiovascular Disease: Common Pathophysiology and Treatment-Emergent Toxicity

**DOI:** 10.1016/j.jaccao.2025.05.003

**Published:** 2025-06-17

**Authors:** Malak El-Rayes, Inbar Nardi Agmon, Christopher Yu, Nichanan Osataphan, Helena A. Yu, Andrew Hope, Adrian Sacher, Anthony F. Yu, Husam Abdel-Qadir, Paaladinesh Thavendiranathan

**Affiliations:** a Department of Medicine, Division of Cardiology, Centre intégré de santé et de services sociaux de Laval, Hôpital Cité de la Santé, Laval, Québec, Canada; b Department of Medicine, Université de Montréal, Montréal, Québec, Canada; c Department of Medicine, Division of Cardiology, Ted Rogers Program in Cardiotoxicity Prevention, Peter Munk Cardiac Center, Toronto General Hospital, University Health Network, University of Toronto, Toronto, Ontario, Canada; d Division of Cardiology, Department of Internal Medicine, Faculty of Medicine, Chiang Mai University, Chiang Mai, Thailand; e Department of Medicine, Memorial Sloan Kettering Cancer Center, New York, New York, USA; f Department of Medicine, Weill Cornell Medical College, New York, New York, USA; g Department of Radiation Oncology, University of Toronto and Radiation Medicine Program, Princess Margaret Cancer Centre, University Health Network, Toronto, Ontario, Canada; h Department of Medicine, Division of Medical Oncology, Princess Margaret Cancer Center, University Health Network, University of Toronto, Toronto, Ontario, Canada; i Women's College Hospital (WCH), Toronto, Ontario, Canada

**Keywords:** cardio-oncology, cardiovascular disease, chemotherapy, lung cancer, radiation therapy, targeted therapy

## Abstract

Among patients with cancer, those with lung cancer have the highest prevalence of pre-existing cardiovascular disease (CVD) and the highest risk of cardiovascular events postdiagnosis. This is driven by shared risk factors, particularly smoking and socioeconomic factors, and common biology. Furthermore, multimodality therapies for lung cancer, including surgery, radiation, chemotherapy, immunotherapy, and targeted therapy, are associated with CVD. Improvements in prevention, screening, and therapy for lung cancer have led to improved cancer survival, increasing the relevance of CVD for overall survival and quality of life. This review provides an overview of lung cancer and its treatment and discusses drivers of CVD, risk assessment, surveillance, prevention, and treatment strategies.

## Highlights

• There are important shared risk factors between lung cancer and CVD, which are increasingly relevant as lung cancer survival improves.
• Among patients with cancer, those with lung cancer have the highest prevalence of pre-existing CVD and are at highest risk of cardiovascular events after diagnosis.
• Treatments used for lung cancer treatment that can contribute to CVD include radiation therapy, chemotherapy, targeted therapy, VEGF antibodies, and immunotherapy.
• Important knowledge gaps remain with respect to risk stratification, prevention, surveillance, and treatment of CVD in patients receiving therapy for lung cancer.

Lung cancer is the second most common cancer after prostate cancer in men and breast cancer in women,^1^^,^^2^ with an estimated incidence of 265,000 cases per year in Canada and the United States combined. Lung cancer is the leading cause of cancer death globally, with 1 in 5 patients dying secondary to this malignancy.^1^^,^^2^

Lung cancer incidence varies internationally, influenced by smoking trends, environmental exposures, and genetics.^3^ The incidence in most economically developed countries peaked in men in the 1980s and women in the 1990s, reflecting trends in tobacco exposure. In emerging economies, the incidence correlates with economic development, tobacco use, air pollution, and exposure to environmental carcinogens (biomass fuels, asbestos, arsenic, and radon), chronologically peaking later than in economically developed countries. In low-income countries, the incidence is highly variable and is linked to smoking patterns and other competing causes of death.^3^

Among persons who have never smoked (<100 cigarettes in their lifetime), lung cancer occurs more frequently in women and individuals of Asian ancestry.^4^ With declining smoking rates, there is an increasing proportion of cancers related to actionable genetic alterations. Younger patients (age <40 years) with lung cancer are more likely to have an actionable genetic alteration.

Lung cancer classification according to histopathologic subtyping of biopsy specimens^5^ and resected tissue provides valuable information for prognosis, subsequent testing for specific genetic alterations, treatment choices, and patient counseling.^6^

Lung cancer is primarily classified into non–small cell lung cancer (NSCLC), accounting for approximately 85% of cases, and small cell lung cancer (SCLC), accounting for the remaining 15%.^5^ NSCLC can be subdivided into adenocarcinoma (78% of NSCLC), squamous cell carcinoma (18% of NSCLC), and other less common types such as adenosquamous carcinoma and large cell carcinoma.^6^ Once NSCLC is diagnosed, imaging modalities are used to categorize patients into stages I to IV (with further subdivisions) using the TNM staging system, with potential for cure associated with nonmetastatic disease.^6^^,^^7^ In addition to staging, testing for tumor programmed death-ligand 1 (PD-L1) expression via immunohistochemistry is indicated to guide treatment with anti–programmed cell death protein 1 or anti–PD-L1 antibodies (immune checkpoint inhibitors [ICIs]).^6^^,^^8^ NSCLC is further classified based on the presence of actionable genetic alterations, which influence tumor growth and invasiveness, with some alterations (eg, *EGFR* and *ALK*) conferring a better prognosis.^9^ Among all subtypes, adenocarcinoma is most frequently associated with specific genetic alterations, which are potential targets for therapy.^6^^,^^9^ Broad next-generation sequencing for oncogenic alterations, including *EGFR*, *ALK*, *KRAS*, *ROS-1*, *BRAF*, *NTRK, RET*, *MET* exon 14 skipping, and *ERBB (HER2)*, and other actionable drug targets are used to further tailor therapy in NSCLC.^8^

SCLC, strongly associated with cigarette smoking, is aggressive, metastasizes early (especially to the brain), and generally confers poor survival.^10^^,^^11^ The main classification is into limited-stage (disease confined to one hemithorax within a tolerable radiation field) vs extensive-stage (disease beyond the ipsilateral hemithorax, including malignant pleural or pericardial effusion or hematogenous metastases) disease, with two-thirds of patients diagnosed with the latter.^11^

In summary, lung cancer is the leading cause of cancer death globally, and the 2 major clinicopathologic subtypes are NSCLC (which accounts for 85% of the disease) and SCLC. Further subtyping based on histologic characteristics, staging, PD-L1 expression, and the presence of specific actionable genetic alterations, provides information about prognosis and guides treatment.

## General Treatment Principles

### Non-Small Cell Lung Cancer

#### Nonmetastatic disease

##### Stages I and II

**Figure 1:**
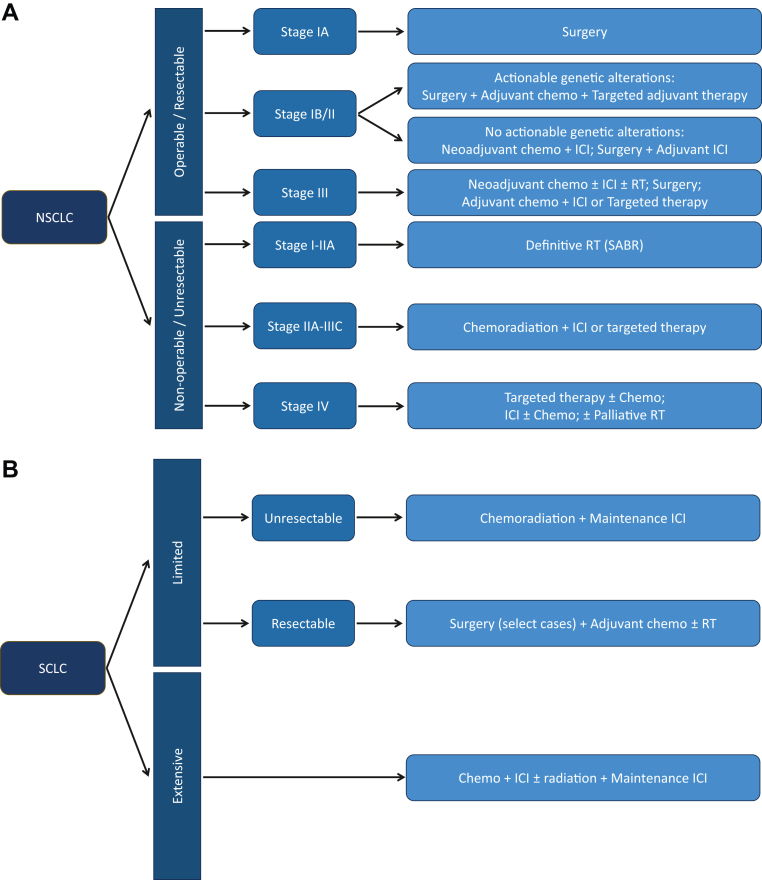
Overview of Treatment Options in Lung Cancer (A) Simplified representation of treatment scheme for non–small cell lung cancer (NSCLC). All stages have further subdivisions that are not presented here. The choice of targeted therapies depends on the presence of actionable genetic alterations and resistance mutations. (B) Simplified representation of the treatment scheme for small cell lung cancer (SCLC). Operable limited stage lung cancer is very rare, and surgery may be considered in select cases. The graphic represents general concepts only: further details are available at the American Society of Clinical Oncology/National Comprehensive Cancer Network^8^^,^^11^^,^^17^, ^18^, ^19^^,^^21^ guidelines. The figure represents contemporary treatment options as of 2025. chemo = chemotherapy; ICI = immune checkpoint inhibitor; RT = radiation therapy; SABR = stereotactic ablative body radiation therapy.

When feasible, surgical resection remains the most effective option for cure in stages I and II (and some IIIA tumors, as discussed in the following section), with preference for lobectomy (anatomical lobar resection) in the majority of patients or segmentectomy (anatomical sub-lobar resection) in very-early-stage disease.^8^^,^^12^ Adjuvant radiation therapy is used in patients with positive surgical margins to reduce the risk of local recurrence. When surgery is undertaken, the integration of neoadjuvant or adjuvant therapies such as platinum-based chemotherapy, ICIs, and targeted therapies in oncogene-driven tumors can significantly enhance survival^8^^,^^13^, ^14^, ^15^, ^16^, ^17^ (Figure 1A). These additional therapies are used in operated patients with disease more advanced than stage IA. For medically inoperable patients (ie, patients with underlying medical conditions that increase surgical risk) with stage I or IIa disease (lymph node–negative tumors <5 cm), radiation therapy, particularly stereotactic ablative body radiation therapy (also known as stereotactic body radiation therapy), is an effective alternative.^8^

##### Stage III

Patients with technically resectable stage IIIa disease are treated with perioperative chemoimmunotherapy and perioperative chemotherapy with adjuvant targeted therapies in oncogene-driven tumors (Figure 1A).^8^^,^^17^ Patients with superior sulcus tumors (Pancoast tumors) receive preoperative chemoradiation followed by adjuvant chemoimmunotherapy, or adjuvant targeted therapy.^8^

Treatment of unresectable stage III NSCLC includes definitive chemoradiation and consolidation ICIs or targeted therapy in the presence of a targetable mutation.^8^

#### Metastatic NSCLC

For metastatic disease with targetable mutation, patients receive targeted therapy, either as an initial or second-line treatment (Figure 1A).^8^^,^^18^ The primary challenge with targeted treatment is the development of resistance mutations, prompting investigations into newer generations of targeted treatment, bispecific antibodies, and/or combination strategies with chemotherapy and ICIs.^6^^,^^18^

For patients without actionable genetic alterations, ICIs are used as first-line therapy if PD-L1 expression is high (≥50% staining of tumor cells) either alone or with platinum-based chemotherapy.^8^^,^^19^ When PD-L1 expression is <50%, a combination of chemotherapy (usually platinum based) and an ICI is recommended as the front-line treatment.

In patients with brain metastases, stereotactic radiation surgery can be used if technically suitable in terms of volume and location.^8^ Some targeted agents also have excellent brain penetration and can effectively treat brain metastases. In patients with large numbers of brain metastases, whole-brain or hippocampal-sparing, whole-brain radiotherapy could be considered with or without resection of large lesions causing edema.^8^

### Small Cell Lung Cancer

#### Limited-Stage SCLC

In limited-stage disease, systemic chemotherapy is combined with early radiation therapy ideally delivered with the first or second cycle of chemotherapy, with recent data supporting the addition of ICIs^11^^,^^20^^,^^21^ (Figure 1B). In patients without disease progression after chemoradiation, as shown in the ADRIATIC (Study of Durvalumab + Tremelimumab, Durvalumab, and Placebo in Limited Stage Small-Cell Lung Cancer in Patients Who Have Not Progressed Following Concurrent Chemoradiation Therapy) trial, the addition of durvalumab (a PD-L1 monoclonal antibody) led to significantly longer overall survival and longer progression-free survival.^20^^,^^21^ For those rare patients with resectable, limited-stage SCLC, surgery may be considered in select cases (very limited stage or uncertain subtype), followed by adjuvant chemotherapy with or without mediastinal radiation.^11^^,^^21^ Chemotherapy regimens in both scenarios usually are platinum based. In addition, prophylactic cranial irradiation is considered for patients with limited-stage disease due to the high frequency (>50% of patients) of brain metastases.

#### Extensive-Stage disease

Initial treatment includes platinum-based chemotherapy and ICI, followed by maintenance ICI^11^^,^^21^ (Figure 1B). Thoracic radiation therapy and prophylactic cranial irradiation improves survival in patients with extensive-stage disease.^22^ In some jurisdictions, prophylactic cranial radiation is replaced with frequent magnetic resonance imaging monitoring and focal treatments with stereotactic radiosurgery.^21^^,^^23^

### Survivorship in the Modern Era of Treatment

Although lung cancer remains the leading cause of cancer death worldwide,^3^ there have been improvements in population mortality in the last 20 years.^24^ Population mortality from SCLC has decreased, mainly due to reduced incidence of the disease related to reduced smoking.^24^ However, 5-year survival in SCLC relative to patients without disease remains low at 7% in all-comers.^10^ In contrast, NSCLC survival has improved with the use of immunotherapy and targeted therapies in oncogene-driven disease^8^^,^^24^ and advances in radiation therapy and screening, with a current 5-year survival rate of 28% for all stages combined (vs 23.3% in 2014),^25^ 65% for earlier stages, and up to 80% in patients with stage I disease.^6^^,^^10^ In metastatic NSCLC treated with immunotherapy, 5-year overall survival ranges from approximately 20% to 30%.^26^ In stage III to IV NSCLC treated with targeted therapy, 5-year overall survival can reach up to 62.5%.^27^

Continuous improvements in overall and lung cancer mortality are anticipated with widespread screening programs,^28^ decreasing rates of smoking in high-income countries, and smoking cessation interventions.^29^^,^^30^ Unfortunately, these survival trends may not be seen in lower income countries due to obstacles in implementing screening programs in these areas; these obstacles include prohibitive cost, limited medical infrastructure, and a greater risk of false-positive findings due to a high prevalence of granulomatous disease (which decreases the net clinical benefit of screening).^3^ In these scenarios, stricter control of tobacco use and environmental factors (air pollution, exposure to radon, biomass fuels, and asbestos) are needed to reduce the burden and mortality related to lung cancer.^3^

As lung cancer prognosis improves, the impact of incident cardiovascular disease (CVD) on morbidity and mortality becomes more apparent. Among patients with cancer, those with lung cancer are at particularly high risk of cardiovascular events.^31^ Although the impact of these cardiovascular events is greater among patients with earlier stage disease and better overall prognosis, these events may also be relevant in some patients with advanced-stage disease who are eligible for targeted therapy. With overall improvement in lung cancer prognosis and the use of cardiotoxic therapies in earlier stage disease, cardiovascular risk mitigation strategies are an integral part of lung cancer management.^32^

### Summary

• Treatment of NSCLC depends on the stage of disease and feasibility of surgical resection (based on patient and tumor characteristics). Definitive treatment includes surgery and/or radiation with or without the addition of chemotherapy and immunotherapy or targeted therapies. In the metastatic setting, treatments include platinum-based chemotherapies, ICIs, targeted therapies, and radiation therapy.
• Most patients with limited-stage SCLC are treated with chemo-radiotherapy, and ICI in patients without disease progression after chemoradiation. The more common diagnosis of extensive stage SCLC is treated with chemotherapy (platinum based), ICIs, and radiation.
• Mortality from lung cancer has decreased with implementation of screening programs, smoking cessation, and better therapies.

## Intersection of Lung Cancer and CVD

### Association Between CVD and Lung Cancer

Cancer and CVD share multiple risk factors, including obesity, hypertension, diabetes, and most notably tobacco smoking.^33^ Socioeconomic factors (income level, educational attainment, employment status, and environmental factors), which are associated with CVD,^34^ are important predictors of lung cancer incidence and mortality.^35^^,^^36^ Finally, shared biology, including chronic inflammation and oxidative stress, plays an important role in the development of CVD and cancer growth and progression.^33^ Cancer itself, through various mechanisms, including secretion of inflammatory factors, metabolic alterations, and cachexia/muscle wasting, can promote the development of CVD.^37^

A recent retrospective study of 894,934 patients with multiple cancers showed that coronary artery disease (CAD) was most prevalent in patients with lung cancer (21% of the lung cancer cohort).^31^ After diagnosis, among the cancer types, patients with lung cancer had the highest adjusted HR for incident major adverse cardiovascular events (MACE; defined as myocardial infarction [MI], stroke, unstable angina, or heart failure) relative to breast cancer (HR: 2.67; 95% CI: 2.60-2.74).^31^ Another population-based study of 21,534 patients linked a new diagnosis of thoracic cancer (primarily lung) to an increased risk of incident stroke (HR: 2.54; 95% CI: 2.39-2.69), heart failure (HR: 3.11; 95% CI: 2.94-3.29), MI (HR: 1.60; 95% CI: 1.41-1.82), and cardiovascular mortality (HR: 1.87; 95% CI: 1.71-2.05), compared with patients without cancer after adjustment for cardiovascular risk factors.^38^ In the United Kingdom Biobank cohort of 478,756 participants who were free of CVD at baseline, patients with lung cancer were more than twice as likely to develop incident CVD (composite of coronary heart disease, heart failure, and stroke) compared with matched control subjects without lung cancer (HR: 2.27; 95% CI: 1.26-4.11).^39^ On the corollary, in the same biobank with 455,804 participants without lung cancer at baseline, CVD was an independent risk factor for incident lung cancer compared with patients without CVD (HR: 1.5; 95% CI: 1.31-1.71). Patients with >2 types of CVD were at the highest cumulative risk of developing lung cancer.

Having both lung cancer and CVD is associated with a poor prognosis. In patients with NSCLC (stages I-IIIB), comorbid CVD, including heart failure, cardiac arrhythmias, and MI, either present 6 months before cancer diagnosis or during the first 6 months of follow-up, was associated with reduced overall survival.^40^ Similarly, patients with pre-existing CVD who develop lung cancer are at increased risk of lung cancer mortality (HR: 1.95; 95% CI: 1.50-2.55) compared with patients without CVD,^41^ likely driven by limited cardiovascular reserve and limitations in cancer treatment selection and tolerance.^40^^,^^42^ In support of the latter, in 20,689 patients diagnosed with NSCLC and SCLC, pre-existing CVD was linked to a reduced probability of receiving chemotherapy (OR: 0.53; 95% CI: 0.48-0.58), surgery (OR: 0.56; 95% CI: 0.44-0.7), and radiation therapy (OR: 0.76; 95% CI: 0.7-0.82).^42^

### Summary

• Lung cancer and CVD share common risk factors, with smoking being the most important.
• Patients with lung cancer have a high prevalence of CVD and are at high risk of incident cardiovascular events. This is associated with worse lung cancer outcomes.
• Patients with CVD are at higher risk of lung cancer than patients without CVD.

## Cardiotoxic Effects of Lung Cancer Therapy

In addition to shared risk factors and biology, lung cancer therapies can contribute to CVD, as discussed here.

### Surgery

Among noncardiac and nonvascular surgeries, thoracic surgery carries the highest risk of perioperative major adverse cardiovascular and cerebrovascular events, with an incidence of 6.5%, representing a 2-fold higher risk than general surgery (OR: 2.07; 95% CI: 2.03-2.11).^43^ Specifically, OR was 3.78 (95% CI: 3.61-3.97) for perioperative stroke, 1.69 (95% CI: 1.63-1.75) for perioperative MI, and 2.02 (95% CI: 1.98-2.07) for perioperative all-cause mortality (Central Illustration).

**Central Illustration:**
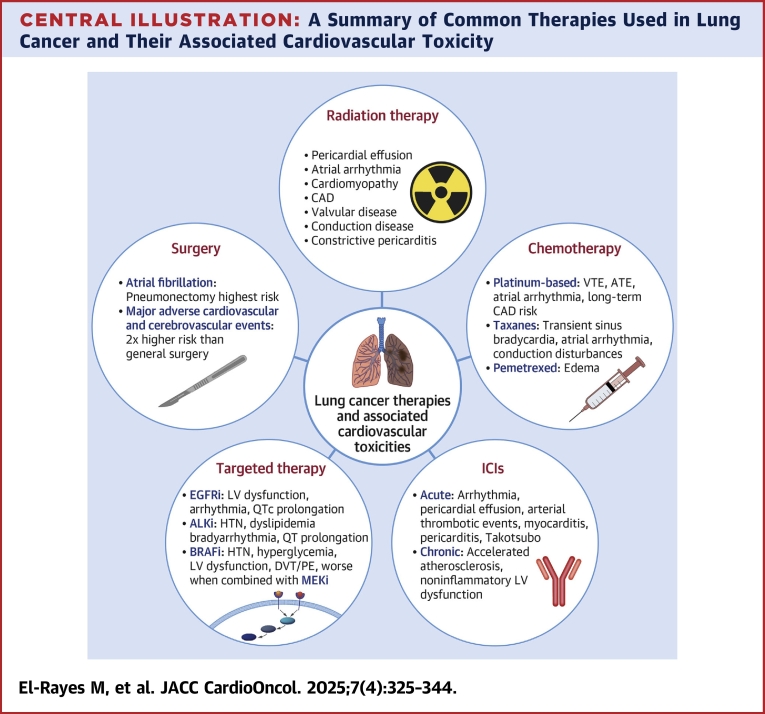
A Summary of Common Therapies Used in Lung Cancer and Their Associated Cardiovascular Toxicity AF = atrial fibrillation; ALKi = anaplastic lymphoma kinase; ATE = arterial thromboembolism; BRAFi = V-Raf murine sarcoma viral oncogene homolog B inhibitor; CAD = coronary artery disease; DVT = deep vein thrombosis; EGFRi = epidermal growth factor receptor inhibitor; HTN = hypertension; ICIs = immune checkpoint inhibitors; LV = left ventricular; MACCE = major adverse cardiovascular and cerebrovascular events; MEKi = mitogen-activated extracellular signal-regulated kinase inhibitor; MI = myocardial dysfunction; PE = pulmonary embolism; SVT = supraventricular tachycardia; VTE = venous thromboembolism.

Atrial fibrillation (AF) is the most frequent cardiovascular event after thoracic surgery, with incidence in one retrospective study of 23.2% post-pneumonectomy, 6.6% post-lobectomy, and 1.4% post-segmentectomy.^44^ In multivariable analysis, postoperative AF (HR: 16.9; 95% CI: 1.8-159.7; *P* = 0.014) was an independent risk factor for perioperative death. Postoperative AF is believed to stem from substantial physiological changes linked with pneumonectomy, including an increase in pulmonary artery pressure, mechanical stretch and increased pressure on pulmonary veins, and increased myocardial strain.^45^ Risk factors for AF include older age, larger left atrial diameter, and a lower left ventricular ejection fraction.^44^^,^^46^

### Radiation

Radiation therapy can cause cardiovascular toxicity (Central Illustration) through various mechanisms. Radiation causes endothelial injury contributing to a pro-inflammatory state.^47^ This results in further damage to blood vessels via oxidative stress, production of reactive oxygen species, and release of cytokines that disrupt the integrity of DNA strands.^47^ The pathophysiological consequences include vessel wall rupture, aggregation of platelets, thrombosis, and fibrosis of the injured intima resulting in vessel stenosis and the development of accelerated atherosclerosis. Also, this pro-inflammatory state leads to the release of growth factors such as tumor necrosis factor, IL-6, IL-8, pro-fibrotic cytokines, fibroblast growth factor, platelet-derived growth factor, and transforming growth factor-β.^48^ Persistent release of these factors influences the myocardial microvasculature affecting oxygen supply demand mismatch and resulting in cardiomyocyte death.^49^ In addition, this inflammatory state results in a greater number of myofibroblasts, collagen deposition, and impaired myocyte contractile function.^47^ Inflammation and microvascular injury also contribute to pericardial inflammation and fibrosis. Clinically, this presents as diastolic dysfunction and/or restrictive cardiomyopathy, conduction abnormalities, AF, and constrictive pericarditis.^49^ Lastly, cardiac valves (especially the aortic valve) are affected by radiotherapy, likely related to fibrosis.^50^ Suggested risk factors include the cardiac radiation exposure (mean heart dose [MHD]) and in particular substructure doses, vascular radiation doses, left atrial and ventricular enlargement on radiation therapy planning scan, coronary artery calcification (CAC) on formal or informal assessment, the presence of cardiovascular risk factors, and pre-existing CVD.^51^, ^52^, ^53^, ^54^ Patients with lung cancer are more prone to the cardiotoxic effects of radiation therapy than other malignancies (eg, lymphoma or breast cancers) as the radiation dose is often higher (commonly >50 Gy), patients tend to be older with more cardiovascular comorbidities,^52^ and the anatomical location of the tumor and associated lymph nodes often make avoiding cardiac structures challenging.

In a recent retrospective study of 478 patients with NSCLC (median age 70 years) undergoing curative intent radiation, 16% experienced MACE (atrial arrythmia, heart failure, and MI) with a median time to event of 16.3 months and a median MHD of 7 Gy.^52^ Higher pre-existing cardiac disease burden (coronary heart disease, arrythmia, or heart failure) was associated with an increased cumulative incidence of MACE (55% [95% CI: 12%-20%] vs 16% [95% CI: 35%-71%]; *P* < 0.001). Recent NSCLC cancer studies have shown that the MHD can be predictive of MACE (cardiac death, unstable angina, MI, coronary revascularization, heart failure hospitalization, or urgent visit) and mortality, with each additional gray unit in MHD being associated with a 5% increase risk of MACE.^55^^,^^56^ In a retrospective study of 701 patients with NSCLC treated with radiation therapy (either curative chemoradiation, neoadjuvant or adjuvant chemoradiation, or radiation), those with high left anterior descending coronary artery (LAD) exposure (≥10% of LAD volume receiving 15 Gy) had the highest incidence of MACE regardless of MHD.^57^ Furthermore, the same authors have suggested that ≥1% of the left ventricular volume receiving >5 Gy increased the 1-year MACE risk by nearly 5% in patients with NSCLC.^58^ These findings suggest that knowing cardiac substructure dosing in addition to MHD may help better identify future cardiac events.^58^ Furthermore, a prediction model incorporating pre-existing risk factors (hypertension and coronary heart disease) and LAD exposure can be used to predict the risk of MACE.^59^

Cardiotoxicity in patients with lung cancer receiving radiation therapy occurs earlier than in other malignancies, with 10% to 22% of patients with locally advanced NSCLC developing it within 2 years.^51^^,^^53^ This underscores the importance of optimizing cardiovascular risk factors and reducing radiation dose to the heart through radiation therapy planning and using techniques such as intensity-modulated radiation, active respiratory motion management, and particle beam therapy (which includes proton beam therapy).^60^, ^61^, ^62^

### Systemic Therapies

#### Platinum-Based therapies

Cisplatin and carboplatin are platinum-containing chemotherapeutic agents that are key treatments for both NSCLC and SCLC. Their use, particularly cisplatin, is associated with an increased risk of venous and arterial thromboembolic events, with an incidence as high as 18.1%.^63^ The most common events are deep vein thrombosis and pulmonary embolism, but MIs and cerebrovascular infarctions have also been reported (Table 1).^63^^,^^64^ The heightened risk persists during treatment and raises long-term cardiovascular risk in survivors, as seen in testicular cancer survivors (20-year absolute risk of up to 8%).^65^ In patients with lung cancer, platinum-based treatment is also associated with more complex anatomical CAD^66^ and an increased risk of arrythmias, particularly AF (Table 1).^67^

**Table 1:** Summary of Common Lung Cancer Therapies and Their Risk of Both Acute and Long-Term CV Toxicities

Agent	CV Toxicity	Estimated Incidence	Suggested Surveillance	Gaps in Knowledge
All potentially cardiotoxic therapies	Any		Pretreatment: In patients receiving potentially cardiotoxic cancer therapy, treating physicians should obtain history of CVD (eg, MI, HF, arrythmias) or CV symptoms (eg, chest pain, shortness of breath, palpitations), and document heart rate and BP. Consider lipid profile, random glucose, and HbA_1c_ testing During treatment: If suspicious CV symptoms occur, consider prompt physical examination and relevant investigations (eg, ECG, cardiac biomarkers, echocardiogram). Refer as appropriate Long term: Inform patients of CVD risk. Routine CV risk factor assessment by general practitioners and appropriate testing based on symptoms	Methods to assess CVD risk before or during cancer therapy have limited validation There is lack of knowledge as to how to titrate CVD surveillance during or after cancer therapy based on patient specific risk Data are lacking on the cost-effectiveness of the surveillance strategies for CVD
Thoracic surgery^43^^,^^44^	Atrial fibrillation		Presurgery: ▪ Assess for poorly controlled CV risk factors and pre-existing CVD ▪ Review medications that may increase bleeding (eg, anticoagulant agents, antiplatelet agents) ▪ Assess for implanted cardiac devices that may be affected (eg, pacemaker, ICD) During/postsurgery: ▪ Monitor and manage BP and electrolyte disturbances	Whether primary prevention strategies can reduce the risk of atrial fibrillation/MACCE is unclear
	Pneumonectomy			
	Lobectomy			
	Segmentectomy			
	MACCE (stroke, MI)			
Chest radiation^49^^,^^51^, ^52^, ^53^^,^^56^^,^^138^	Pericardial effusion	/ a	▪ Assess for implanted cardiac devices that may be affected (eg, pacemaker, ICD) ▪ No routine surveillance during treatment, consider symptom-driven investigations ▪ Long-term monitoring: echocardiography and noninvasive screening for CAD starting at 5 years after radiation to those exposed to >15 Gy MHD of radiation	It is uncertain whether specific cardiac substructure that is exposed to radiation should guide subsequent surveillance strategy
	Atrial arrythmias	/ a		
	HF			
	Valvular disease			
	MI	/ a		
	Conduction abnormalities			
	Constrictive pericarditis			
Platinum-based therapies^63^^,^^65^^,^^67^	Venous/arterial thrombosis		▪ No routine surveillance during treatment, symptom-driven investigations	Whether patients treated at young age should have coronary assessment later in life, regardless of symptoms
	Long-term risk for CAD			
	Atrial arrythmias			
Taxanes^67^, ^68^, ^69^	Transient sinus bradycardia		▪ Check heart rate at clinic visits	Whether certain patient characteristics are associated with higher risk of bradycardia and arrythmias is unclear
	Conduction disturbances (AV nodal blocks, LBBB)			
	Atrial arrythmias			
Pemetrexed^71^	Peripheral edema (noncardiac origin)		▪ Assess for peripheral edema at clinic visits ▪ History and physical examination to rule out cardiac origin	
EGFR inhibitors^73^^,^^75^^,^^76^	Decrease >10% in LV function	b	▪ A baseline ECG in patients who receive osimertinib with documentation of QTc. Periodic monitoring with ECG and electrolytes in patients at high risk of QT prolongation^c^ ▪ Baseline echocardiogram and periodic echocardiograms in patients with CV risk factors who receive osimertinib monotherapy and in all patients who receive osimertinib with pemetrexed/platinum-based chemotherapy	The frequency of follow-up echocardiograms with osimertinib or the need for echocardiograms with other EGFR inhibitors is not defined
	HF	b		
	QT prolongation	b		
	Atrial fibrillation	b		
	VTE			
	MI			
	Ventricular arrythmias			
	Pericardial effusion			
	Conduction abnormalities			
ALK inhibitors^73^^,^^79^, ^80^, ^81^, ^82^	Hypertension	d	▪ A baseline ECG is recommended in all patients with documentation of QTcF ▪ Monitor heart rate and BP regularly ▪ With lorlatinib and brigatinib: home BP monitoring may be considered ▪ Repeated ECG to assess QTcF may be considered 4 weeks after starting treatment ▪ With lorlatinib: initiate or increase the dose of lipid-lowering agents in patients with dyslipidemia. Monitor lipid levels at baseline, then 1 and 2 months after starting treatment, and periodically thereafter	The timing and frequency of lipid surveillance with lorlatinib or the need for ECG surveillance with other drugs in the class are unclear
	Dyslipidemia	e		
	Bradycardia and conduction disturbances	/f		
	QT prolongation	/f		
	HF			
	Arrythmias			
BRAF/MEK inhibitors^73^^,^^85^	Hypertension	g	▪ Monitor BP at clinic visits ▪ Baseline ECG and echocardiogram should be considered in all patients receiving combination therapy ▪ Monitor and correct electrolytes before and during treatment ▪ Repeated echocardiography could be considered after 4 weeks and subsequently every 2-3 months with combination therapy ▪ Monitor serum glucose levels in patients with pre-existing diabetes who are treated with MEK inhibitors	Whether TTE surveillance is required when a single agent is used is unclear
	Hyperglycemia	h		
	LV dysfunction	/g		
	PE	/g		
	QT prolongation			
	Conduction disturbances			
VEGF antibodies^86^^,^^90^	Hypertension		▪ Monitor BP frequently with the first cycle and after any dose change, then every 2-3 weeks ▪ Baseline echocardiogram may be considered in patients with pre-existing CVD or prior exposure to potentially cardiotoxic treatment (eg, anthracyclines)	Whether echocardiographic surveillance is useful is unclear
	VTE			
	Arterial thrombotic events (MI, stroke)			
	HF			
ICIs^91^^,^^95^^,^^98^, ^99^, ^100^	Atrial arrhythmias		▪ Baseline troponin and ECG in all patients and consider baseline echocardiography in patients with pre-existing CVD	Whether CV surveillance is required in all patients and the best monitoring strategy and frequency are unclear
	Pericardial effusion			
	HF			
	Arterial thrombotic events (MI, stroke)			
	Myocarditis			
	Pericarditis			
	VTE			
	Takotsubo cardiomyopathy			
	Vasculitis			
	Accelerated atherosclerosis and long-term increased risk for CAD	Unknown		

The table also outlines suggested surveillance strategies and gaps in knowledge. Estimated incidence:  < 1%;  1%-5%;  5%-10%;  >10%.

ALKI = anaplastic lymphoma kinase inhibitor; AV = atrioventricular; BP = blood pressure; BRAFi = V-Raf murine sarcoma viral oncogene homolog B inhibitor; CAD = coronary artery disease; CV = cardiovascular; CVD = cardiovascular disease; ECG = electrocardiogram; HbA_1c_ = glycosylated hemoglobin; ICD = intracardiac defibrillator; ICIs = immune checkpoint inhibitors; LBBB = left bundle branch block; LV = left ventricular; MACCE = major adverse cardiac and cerebrovascular events; MEKi = mitogen-activated extracellular signal-regulated kinase inhibitor; MHD = mean heart dose; MI = myocardial infarction; PE = pulmonary embolism; QTcF = QT corrected for heart rate by Fridericia's cube root formula; TTE = transthoracic echocardiography; VEGF = vascular endothelial growth factor; VTE = venous thromboembolism.

a Incidence varies between reports.

b Higher incidences with osimertinib than with other epidermal growth factor receptor (EGFR) inhibitors.

c Higher risk for QT prolongation: congenital long QTc syndrome, congestive heart failure (HF), electrolyte abnormalities, concurrent QT prolonging medications.

d With lorlatinib/brigatinib treatment.

e With lorlatinib treatment.

f Higher incidence with ceritinib compared with other ALK inhibitors.

g Higher incidence with combination therapy BRAF/MEK inhibitors.

h With MEK inhibitors.

#### Taxanes

Paclitaxel and docetaxel are most frequently used in NSCLC. Taxanes rarely cause cardiotoxicity but have been associated with high rates of transient sinus bradycardia.^68^ Conduction disturbances and atrial arrythmias have also been described (Table 1).^68^^,^^69^ Taxane treatment (more commonly docetaxel) can cause edema through increased capillary permeability and lymphedema.^70^

#### Pemetrexed

Pemetrexed is an antifolate drug that disrupts cell replication. It is frequently used with platinum agents to treat nonsquamous NSCLC. Pemetrexed is rarely linked to cardiovascular toxicities but can cause peripheral edema of noncardiac origin in about 1% of patients due to capillary leakage (Table 1).^71^

#### Targeted treatments

Targeted therapies refer to small molecule inhibitors that target specific actionable genetic alterations identified in lung cancer (Table 1 summarizes the incidence of cardiovascular events).

##### EGFR Inhibitors

The epidermal growth factor receptor (EGFR) is a cell surface receptor that promotes DNA synthesis, cell cycle progression, and proliferation. Somatic mutations in the *EGFR* gene causes its hyperactivation, driving tumor growth. EGFR tyrosine kinase inhibitors (TKIs) such as osimertinib block adenosine triphosphate binding to the receptor, disrupting its activity.^72^ Among EGFR-TKIs, osimertinib has been found through in vitro models to have greater activity against HER-2, which may mediate the greater risk of cardiotoxicity with this drug.^73^

Use of osimertinib is associated with an approximately 5% risk of adverse cardiac events, including left ventricular systolic dysfunction, symptomatic heart failure, supraventricular tachycardia, and QT prolongation.^73^^,^^74^ Other cardiovascular toxicities include AF, MI, stroke, and venous thromboembolism.^74^^,^^75^ A decrease in left ventricular ejection fraction of ≥10% to <50% occurred in 3% to 5.5% of patients in the FLAURA (A Phase III, Double-Blind, Randomized Study of Osimertinib versus Gefitinib or Erlotinib in Previously Untreated Patients with EGFR-Mutated Advanced Non-Small Cell Lung Cancer) and AURA3 (A Phase III, Open Label, Randomized Study of osimertinib versus Platinum-Based Doublet Chemotherapy in Patients with EGFR T790M Positive Advanced NSCLC) trials, with older age and hypertension being risk factors.^76^ In a pharmacovigilance study (U.S. Food and Drug Administration Adverse Events Reporting System), cardiac failure represented 2.3% of reported adverse events due to osimertinib, AF 1.2%, QT prolongation 1.3%, MI 0.7%, and pericardial effusion 0.6%.^75^ Compared with earlier-generation EGFR-TKIs (erlotinib, afatinib, and gefitinib), osimertinib was associated with higher reports of cardiac failure (reported OR [rOR]: 2.2), AF (rOR: 2.1), QT prolongation (rOR: 6.6), MI (rOR: 1.2), and pericardial effusion (rOR: 1.6).^75^ Despite the cardiovascular events associated with osimertinib, this drug has been associated with significant improvement in overall survival in *EGFR*-mutated NSCLC compared with older generation EGFR-TKIs (erlotinib and gefitinib).^77^ As new generations of EGFR-TKIs emerge, further studies are needed to investigate their cardiotoxicities.

##### ALK Inhibitors

A rearrangement in the anaplastic lymphoma kinase (ALK) gene receptor results in constitutive activation of the receptor and pathologic cell proliferation. The ALK inhibitors (eg, crizotinib, ceritinib, alectinib, lorlatinib, brigatinib) are TKIs that bind to the hyperactivated ALK receptor and silence its mutated activity.^72^

These drugs are mainly associated with bradyarrhythmia and QT prolongation,^78^^,^^79^ with other side effects, including hypertension, hyperglycemia, and dyslipidemia (Table 1).^78^^,^^80^^,^^81^ In a meta-analysis of randomized controlled trials in patients with advanced NSCLC, crizotinib was associated with a 9% incidence of bradycardia, and alectinib with 4%, during a mean follow-up of 1.26 and 1.3 years, respectively.^79^ In a pooled analysis, of clinical trials in which patients received ALK-TKIs for advanced NSCLC, the incidence of QTc interval prolongation with ceritinib, crizotinib, alectinib, and lorlatinib was 6.7%, 2.4%, 3.2%, and 0%.^82^ Grade ≥3 hypertriglyceridemia (16%-20%) and hypercholesterolemia (15%-16%) were common for lorlatinib and were rarely reported in other ALK-TKIs. A pharmacovigilance study using the VigiBase database found that ALK-TKIs were associated with increased odds of conduction disease (rOR: 12.95; 99% CI: 10.14-16.55) and QT prolongation (rOR: 5.16; 99% CI: 3.92-6.81) compared with EGFR-TKIs and BRAF inhibitors combined.^73^ Crizotinib had the highest risk of for both conduction disease (rOR: 1.75; 99% CI: 1.30-2.36) and QT prolongation (rOR: 1.91; 99% CI: 1.22-3.00) compared with other ALK-TKIs.

##### BRAF Inhibitors

The V-Raf murine sarcoma viral oncogene homolog B (BRAF) gene encodes the BRAF protein, which plays a role in cell differentiation and proliferation. A somatic mutation in the *BRAF* gene causes hyperactivation of the BRAF protein, resulting in overstimulated proliferation of the mutated cells. The BRAF-TKIs (eg, dabrafenib, trametinib, encorafenib) inhibit the mutated protein or interfere along its action cascade.^83^ These drugs are associated with hypertension, left ventricular systolic dysfunction, and venous thromboembolism/pulmonary embolism.^84^ Importantly, combined therapy with mitogen-activated extracellular signal-regulated kinase (MEK) inhibitors (small molecules that interfere with the same signaling cascade)^83^ increases the risk of pulmonary embolism (RR: 4.26; 95% CI: 1.23-15.45), cardiac dysfunction (RR: 3.72; 95% CI: 1.74-7.95), and arterial hypertension (RR: 1.49; 95% CI: 1.12-1.98) compared with BRAF inhibitor monotherapy.^85^

#### Other targeted therapies

Cardiotoxicity data for other targeted therapies such as ROS1 inhibitors (eg, crizotinib, entrectinib, lorlatinib, repotrectinib), MET inhibitors (eg, capmatinib, tepotinib), RET inhibitors (eg, selpercatinib, pralsetinib), and KRAS G12C inhibitors remain limited, with sporadic case reports suggesting the potential for left ventricular dysfunction and arrythmias.^86^^,^^87^

#### Vascular endothelial growth factor antibodies

Vascular endothelial growth factor (VEGF) antibodies are monoclonal antibodies that bind VEGF, thereby inhibiting tumor blood vessel growth (eg, bevacizumab, ramucirumab).^88^ The main adverse event in patients with advanced NSCLC with these drugs is hypertension (up to 30% with bevacizumab^89^ and 11% with ramucirumab^90^). Other reported cardiovascular events include venous and arterial thromboembolic events, as well as LV dysfunction (Table 1).^89^^,^^90^

#### Immune checkpoint inhibitors

ICIs re-activate T cells, which have been inactivated by tumor cells, to enhance antitumor immune response. Although highly effective in lung cancer treatment, the activated T cells can lead to immune-related adverse events.^91^ In the cardiovascular system, ICI toxicity has been mainly associated with immune-mediated myocarditis, often accompanied by myasthenia gravis (22% presenting with diplopia and/or ptosis) and myositis (24% with myalgias).^92^^,^^93^ Although the incidence in the literature has been variable, and most meta-analyses have reported an incidence <1%,^91^^,^^94^^,^^95^ subclinical cases may be more common due to lack of routine surveillance.^93^^,^^96^ However, the relevance of subclinical cases remains unclear and may not warrant immunosuppressive therapy.^93^ Other acute cardiovascular toxicities include conduction disturbances, arrhythmias, Takotsubo syndrome, acute venous and arterial thrombotic events, pericarditis, pericardial effusion, vasculitis, and pulmonary hypertension.^96^^,^^97^

Incidence of these events based on a recent meta-analysis^95^ and other studies, although subject to limitations, is illustrated in Table 1. A safety meta-analysis reported incidence rates of 3.2 per 1,000 patients for myocarditis, 7.4 for MI, 8.3 for pericardial disease, 8.8 for ischemic stroke, and 8.7 for heart failure during a follow-up range of 3.2 to 32.8 months.^96^ ICIs are associated with a 7-fold increased risk for MI, a 3-fold increased risk for coronary revascularization, and a 4-fold increased risk for ischemic stroke compared with a non-ICI cohort matched by age, CVD history, and cancer type.^98^ Acute cardiovascular adverse events often occur early in treatment, with 81% of myocarditis occurring within the first 3 months and acute vascular events occurring more frequently in the first 6 months than between 6 and 12 months (OR: 3.49; 95% CI: 1.45-8.41).^91^^,^^99^ However, late complications, such as accelerated atherosclerosis, ischemic events, and non-inflammatory left ventricular dysfunction, are being increasingly described.^99^^,^^100^

#### Bispecific antibodies

There is an expanding role for bispecific antibodies in the treatment of lung cancer, with amivantamab (EGFR-MET–bispecific antibody) approved for the treatment of advanced *EGFR*-mutated NSCLC.^101^^,^^102^ These are engineered hybrid antibodies that target 2 antigens in the tumor cell activation pathway.^101^ Amivantamab has been most frequently associated with venous thromboembolism; other cardiovascular events include hypotension, arrhythmia, and pericardial effusion.^103^ Other bispecific antibodies are being investigated in the treatment of lung cancer, including molecules targeting PD1/VEGF in advanced NSCLC^101^ and in combination with EGFR-TKI.^102^ Side effects of these drugs and combinations will be better understood as clinical use expands.^101^

### Summary

• Among noncardiac surgery, thoracic surgery carries a risk of major adverse cardiovascular and cerebrovascular events similar to that of vascular surgery. Treatment of pre-existing CVD should be optimized before undergoing thoracic surgery for lung cancer.
• Radiation therapy–related cardiotoxicity occurs earlier than in other malignancies. Cardiac substructure dosing in addition to MHD may help better predict cardiac events. Predictive models should incorporate underlying risk factors such as hypertension and pre-existing coronary heart disease.
• Systemic therapies are associated with a spectrum of cardiovascular toxicities, including arrhythmias, heart failure, vascular events, hypertension, and myocarditis (Table 1).

## Risk Assessment and Surveillance Strategies

The commencement of lung cancer treatment is an opportune time for cardiovascular risk assessment and prevention.^84^^,^^104^ For patients receiving potentially cardiotoxic therapies, pretherapy evaluation by the treating physician (eg, oncologist or radiation oncologist) should include a cardiovascular history (eg, MI, heart failure, arrhythmias, significant valvular heart disease) and physical examination (eg, blood pressure, heart rate, signs of heart failure or significant murmurs) and assessment for the presence of traditional cardiovascular risk factors (eg, diabetes, smoking, dyslipidemia, lack of physical activity, obesity, smoking) (Table 1).^104^ This may be achieved through standardized templates or patient questionnaires.

Based on this assessment, additional testing and referrals should be considered without delaying cancer treatment in patients without active cardiovascular symptoms (eg, angina, heart failure, palpitations) or high-risk features (eg, poorly controlled arrhythmias, severe left ventricular dysfunction, multi-vessel coronary disease) on initial evaluation.^84^^,^^104^ Optimization of CVD and risk factors should be done before, during, and after cancer therapy, ideally by the treating team and through relevant referrals. Because treatment thresholds and targets for cardiovascular risk factors in cancer patients are not established, decisions should be individualized based on comorbidities, drug interactions, and cancer therapies. Specific cardio-oncology guidelines (where available) for risk assessment and surveillance in lung cancer treatments are summarized in Table 1. A recent expert consensus document and the European Society of Cardiology/International Cardio-Oncology Society cardio-oncology guidelines provide further information about general pretreatment risk assessment in patients with cancer.^84^^,^^104^ However, these recommendations largely represent expert opinion and are based on limited data. Given this limitation, it may be reasonable to make decisions regarding baseline and longitudinal monitoring on a case-by-case basis, taking into account baseline cardiovascular risk, cancer stage and prognosis, and locally available resources.

### Opportunistic Screening

**Figure 2:**
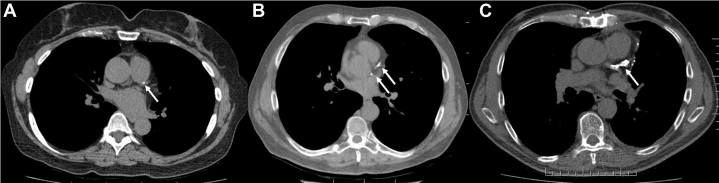
Examples of Incidental Coronary Calcium Identified on Chest CT Scans Examples of mild (A), moderate (B), and severe (C) visual coronary calcium are shown. White arrows point to the location of the coronary calcium, which was in the left anterior descending coronary artery in all 3 patients.

Imaging modalities used for screening, staging, and monitoring of lung cancer should be used as opportunities for CVD risk assessment by identifying CACs.^105^ Although commonly assessed by using dedicated electrocardiogram (ECG)-gated cardiac CT scans, CAC can also be identified and quantified by using non–ECG-gated chest CT scans. Using non-contrast CT images from 1,442 patients undergoing lung cancer screening, Chiles et al^106^ reported adjusted HRs (95% CI) for coronary heart disease death for Agatston scores (modified Agatston method) 1 to 100, 101 to 1,000, and >1,000 of 1.27 (0.69-2.53), 3.57 (2.14-7.48), and 6.63 (3.57-14.97), respectively, compared with those with a zero score. Furthermore, using visual assessment (Figure 2) to categorize coronary calcium as mild, moderate, or severe, the adjusted HRs for coronary heart disease death was 2.09 (1.30-4.16), 3.86 (2.02-8.20), and 6.95 (3.73-15.67) compared with no calcium. Similar associations were seen with calcium score and visual assessment and all-cause mortality. This shows that if the radiologists are unable to provide a calcium score in their chest CT report, simple visual description may still be useful.

Scans used in radiation therapy planning can also identify CAC. A recent study using artificial intelligence–based algorithms showed an association between the presence of CAC on these scans and increased mortality.^107^ Although there are no specific data on treatment guidance in patients with cancer, general recommendations are to consider moderate-intensity statins in those with CAC scores of 1 to 100 or mild calcium on visual assessment, moderate- to high-intensity statins and aspirin 81 mg daily in those with CAC scores of 101 to 299 or moderate visual calcium, and high-intensity statins and aspirin 81 mg daily in those with a CAC score ≥300 or severely increased visual calcium.^108^^,^^109^ In patients with cancer, however, careful consideration is needed with respect to bleeding risk before consideration of aspirin therapy. In those at elevated risk, there may be net harm with the use of aspirin for primary prevention.^110^

### Surgery

Preoperative evaluation should focus on patients with significant cardiovascular history, risk factors (including prior cardiotoxic therapy), or symptoms.^84^ Treatment of cardiovascular risk factors and pre-existing CVD should be optimized before thoracic surgery.^111^ Smoking cessation counseling is crucial before lung cancer surgery, as early smoking cessation decreases the odds of major complications and mortality in the peri-operative period.^112^ Exercise testing for the evaluation of a patient's function/exercise capacity may provide further risk stratification before lung surgery, as it has been associated with the perioperative risk for morbidity and mortality.^113^ Strategies to prevent postoperative AF include ensuring hemodynamic stability before surgery, correcting electrolyte imbalances, and continuing beta-blockers if patients are taking these agents.^111^ Limited data suggest that prophylactic amiodarone may be considered for patients at the highest risk for AF such as those having anatomical resection and are aged >65 years,^114^ or for patients with pre-existing paroxysmal AF that is poorly tolerated.

### Radiation Therapy

In addition to assessment of CAC on radiation-planning scans, other suggested baseline investigations for risk stratification include an ECG and echocardiogram.^115^ Prevention of radiation-associated cardiotoxicity involves careful radiation therapy planning and techniques to minimize cardiac radiation exposure, such as intensity-modulated radiation, active respiratory management, image-guided radiation therapy, and particle mean therapy. As an example, active respiratory motion management, which includes deep inspiration breath-holding, flattens the diaphragm while the lung expansion pulls the heart toward the center of the chest, distancing it from the radiation field.^60^, ^61^, ^62^^,^^115^ Optimal management of existing coronary heart disease and comorbid risks such as hypertension is important, as these factors increase the risk of adverse cardiovascular events in those receiving radiation therapy.^52^^,^^59^

### Cardiotoxic Systemic Therapy

Primary prevention of cardiotoxicity involves a thorough pretreatment cardiovascular risk assessment and improved control of CVD and cardiovascular risk factors (Table 1). Given that cancer treatments can affect blood pressure, periodic screening at home or during clinic visits is recommended, especially with TKIs such as ALK inhibitors and BRAF/MEK inhibitors and VEGF antibodies.^84^^,^^85^

### Targeted Treatments

Given the risk of arrhythmias with osimertinib and ALK inhibitors, a baseline ECG, including documentation of QTc, is recommended.^88^ Although there are no data to support ECG monitoring during treatment, periodic ECGs in patients receiving osimertinib who may be a risk of QTc prolongation (eg, those with electrolyte abnormalities, congestive heart failure, unavoidable use of other QTc-prolonging medications, and congenital long QT syndrome) should be considered.^88^ A repeated ECG at 4 weeks after treatment initiation may be considered for patients taking ALK inhibitors, particularly if the baseline ECG is abnormal, in addition to measuring heart rate and blood pressure at clinic visits.^84^^,^^88^^,^^116^ It is important to assess potential drug–drug interactions or the presence of other QT-prolonging treatments in addition to electrolyte monitoring to avoid further risk of QT prolongation and associated arrythmias. Suspicious symptoms such as dizziness and syncope may warrant Holter monitoring.^78^

In patients receiving osimertinib, baseline echocardiography is recommended, and it is unclear whether this practice is required for patients taking older generation EGFR-TKIs.^84^^,^^88^ Serial echocardiography during treatment may be considered in patients with prior CVD or prior cardiotoxicity and those receiving osimertinib in combination with pemetrexed and platinum-based chemotherapy (based on Summary of Product Characteristics package insert).^84^^,^^88^ With the newer EGFR-TKIs, further data are needed to determine the role of follow-up echocardiograms during treatment.

In patients receiving lorlatinib, it is suggested that lipid profiles be assessed at baseline and every 1 to 2 months on therapy until stable, then every 3 to 6 months^88^ (Table 1).

In patients receiving BRAF/MEK inhibitors, a baseline ECG, echocardiography, and assessment of blood pressure should be considered.^117^ During therapy, echocardiography could be repeated at 4 weeks and subsequently every 3 months based on Summary of Product Characteristics package inserts for these drugs. Monitoring blood pressure during treatment should be considered weekly for the first cycle and every 2 to 3 weeks subsequently. During treatment, an ECG for QT monitoring should be considered at 1 month and with any subsequent dose adjustment.

### Immune Checkpoint Inhibitors

Baseline ECG and cardiac troponin assessments are recommended in all patients with consideration for an echocardiogram in those deemed to be at high cardiotoxicity risk (eg, dual ICIs, combination of ICI and cardiotoxic therapy, known CVD, prior toxicity).^84^ This baseline assessment would be relevant to compare abnormalities identified during therapy, especially in patients with nonspecific symptoms with concern for cardiovascular toxicity. Current data are insufficient to support the use of routine cardiac troponin screening during therapy in the absence of symptoms.^88^^,^^118^

### Long-Term CVD Surveillance

As cancer survival improves and potentially cardiotoxic treatments are used in earlier stage disease, long-term cardiac surveillance is increasingly important.^13^, ^14^, ^15^ Lung cancer survivors who have undergone radiation therapy are a particularly high-risk group. Although the greatest risk of CVD occurs in the first year after cancer diagnosis, it remains elevated in the long term (5-10 years).^38,1119^ British data suggest that lung cancer survivors have nearly double the risk of heart failure (HR: 1.95; 95% CI: 1.36-2.79) beyond 5 years compared with matched control subjects.^119^ Follow-up care should include monitoring cardiovascular risk factors, including blood pressure, lipid profiles, and glucose levels, with optimization according to local guidelines. Although there are no specific guidelines for long-term CVD surveillance in lung cancer survivors, the existing societal recommendations following chest radiation therapy, which are applicable to patients with lung cancer, are summarized in Table 2.

**Table 2:** Summary of the Key Recommendations From the Society Position Statements and Guidelines on Cardiac Surveillance After Chest Radiation Therapy

ASCO 2017 Survivors Guideline^139^	ESMO 2020 Position Statement^140^	ICOS 2021 Radiation Therapy Consensus^115^	ESC 2022 Guideline^84^
TTE at 6-12 months after completion of cancer therapy in those with RT ≥30 Gy in which the heart is in the treatment field. If inadequate TTE images, CMR is preferred over MUGA	Annually: History and physical examination In those who are: Symptomatic: TTE annually Asymptomatic: TTE at 5 years’ posttherapy for evaluation of valvular disease and further testing for ischemia. Then at least every 3-5 years thereafter	Annually: Cardiovascular history and examination Review CT imaging for atherosclerotic calcification Optimize cardiovascular risk factors and disease Bilateral blood pressure assessment Assess for signs of SVC obstruction/stenosis TTE at 6-12 months in high-risk patients Every 5 years a TTE and ischemic evaluation for all patients	Annually: Cardiovascular risk assessment with risk factor optimization. This includes lipid profile, HbA_1c_, ECG, and natriuretic peptide TTE imaging based on risk:^a^ Moderate risk: TTE every 5 years High and very high risk: TTE at 1, 3, and 5 years after cardiotoxic cancer therapy, then every 5 years Can also consider noninvasive screening for coronary artery disease in those who received >15 Gy MHD and carotid disease (in those with a history of head/neck radiation therapy) every 5-10 years starting at 5 years after radiation therapy

ACSO = American Society of Clinical Oncology; CMR = cardiac magnetic resonance; ESC = European Society of Cardiology; ESMO = European Society for Medical Oncology; ICOS = International Cardio-Oncology Society; MUGA = multi-gated acquisition; RT = radiation therapy; SVC = superior vena cava; other abbreviations as in Table 1.

a Radiation therapy risk definition: Very high-risk >25 Gy MHD; high risk: 15-25 Gy MHD; moderate risk: 5-15 Gy MHD; and low risk: <5 Gy MHD.

### Summary

• The commencement of lung cancer treatment provides an opportune time for cardiovascular risk assessment.
• Cardiovascular risk assessment could include cardiovascular history and examination, assessment of risk factors, review of available chest CT scans to identify coronary calcification, ECGs, echocardiography, and functional testing as appropriate.
• There is limited specific guidance on the most appropriate routine cardiovascular surveillance during and after lung cancer treatment. Decisions should be driven by patient's baseline cardiovascular risk profile, specific exposure to cardiotoxic treatments, and the development of cardiotoxicities.

## Management of Cardiotoxicity

When cancer therapy–related cardiotoxicity develops, a multidisciplinary patient-centered team discussion is required to decide on the best management.^84^^,^^88^ The risks and benefits of pursuing cancer therapy, presence of alternative treatment options, cancer prognosis, and the need to initiate cardiovascular therapy are among the key considerations. Management of cardiotoxicities in general have been discussed elsewhere recently^84^^,^^88^; the focus here is only on certain lung cancer–specific toxicities.

### Osimertinib-Induced Cardiac Dysfunction

Patients who develop symptomatic cardiac dysfunction with osimertinib can be successfully managed with interruption of therapy and initiation of guideline-directed heart failure therapy.^120^ Re-initiating osimertinib requires a multidisciplinary approach considering the degree of left ventricular dysfunction, available alternative treatments, presence of central nervous system disease, and mutations that may render the tumor less sensitive to other lines of treatment. Although left ventricular ejection fraction is not routinely tracked, it may be reasonable to continue treatment in the setting of mild asymptomatic left ventricular dysfunction given the significant benefit on disease control and overall survival with osimertinib,^78^ with careful surveillance and cardio-oncology involvement.

### Bradycardia on ALK Inhibitors

With alectinib, bradycardia is generally asymptomatic and does not require specific intervention.^121^ For symptomatic sinus bradycardia, dose reduction or switching to an alternative agent may be considered. In rare cases, a permanent pacemaker may be needed to permit continuation of therapeutic doses.

### Lipid Abnormalities With Lorlatinib

Although the long-term impact of lipid abnormalities induced by lorlatinib is unknown, treatment with lipid-lowering agents is generally recommended, especially in the context of good response to cancer therapy.^88^

### ICI-induced Myocarditis

Prompt recognition and initiation of corticosteroids is recommended to reduce MACE and improve outcomes (details are provided elsewhere).^88^ In patients with fulminant myocarditis, high-dose steroids and the addition of a second immunomodulatory agent should be considered.^84^^,^^88^ Gradual tapering of corticosteroids is recommended once there is improvement in cardiac biomarkers, with close clinical and biochemical follow-up.^84^ Re-challenge upon recovery from ICI myocarditis requires a multidisciplinary discussion, considering myocarditis severity, response to therapy, disease stage, and availability of alternative therapies.^84^

### Treatment of Pericardial Effusions in Patients With Lung Cancer

Lung cancer is the most frequent cause of metastatic pericardial effusion.^122^ In addition, pericardial effusion can result from cancer treatments such as radiation and ICIs. In patients with hemodynamic compromise related to pericardial effusion, urgent pericardiocentesis is recommended, with the catheter left in place for 2 to 5 days to prevent recurrence. Intrapericardial treatment after drainage can be considered, although sclerosing and cytotoxic agents have only shown modest success in reducing recurrence. A surgical pericardial window is typically recommended for recurrence, as it reduces the risk of repeat intervention and offers the highest recurrence-free rates compared with simple pericardiocentesis or balloon pericardiotomy in patients with metastatic NSCLC.^122^^,^^123^

### Treatment of Selected CVD in Patients With Concurrent Lung Cancer

In patients with active lung cancer and acute cardiovascular events, treatment decisions must consider the expected magnitude and timeline of benefit from a cardiovascular intervention, the lung cancer prognosis, the impact of interrupted therapy, thrombocytopenia, bleeding risk, and the goals of care.^124^ These issues have been recently discussed in detail in a state-of-the-art review.^124^ An approach to the treatment of heart failure, acute coronary events, AF, and severe symptomatic AS is briefly discussed here given their more common occurrence.

#### Heart Failure

In patients who develop heart failure during cancer treatment, a multidisciplinary team approach is required to ascertain the cause, begin guideline-directed medical therapy, and address continuing or modifying cancer therapy.^84^ The diagnosis of acute heart failure may be particularly difficult as dyspnea can be misinterpreted as related to the lung cancer itself. A high index of suspicion, physical examination findings, use of N-terminal pro–B-type natriuretic peptide levels, and echocardiography may be required. Once heart failure is diagnosed, among those with cancer, patients with lung cancer are at the highest risk of in-hospital (adjusted OR: 2.71; 99% CI: 2.37-3.09) and 1 year (adjusted HR: 1.76; 99% CI: 1.62-1.90) mortality.^125^ Although guideline-directed medical therapy should be initiated at diagnosis, dose reductions may be required due to hypotension, neutropenia, renal failure, and drug interactions.^126^ Furthermore, patients receiving diuretics should have electrolyte monitoring, especially in the context of targeted therapies, as hypokalemia and hypomagnesemia may increase the risk of QTc prolongation and torsade de pointes.^84^^,^^88^ Given that patients with lung cancer are at high risk of underlying CAD,^31^ evaluation of CAD should be considered. Furthermore, cardiac magnetic resonance imaging may be useful in identifying the cause of heart failure. In patients with persistently reduced left ventricular ejection fraction despite guideline-directed medical therapy, cardiac implantable electronic devices may be considered, while balancing prognosis, impact on future imaging for cancer monitoring (eg, magnetic resonance imaging), and implications for interference with radiation therapy.^124^

#### Acute coronary syndromes

In patients with ST-segment elevation MI (STEMI), a primary invasive strategy is generally recommended regardless of cancer staging if consistent with the patient’s goals of care and in the absence of clinical futility.^124^ Patients with lung cancer presenting with STEMI are at high risk of in-hospital mortality (32.9%),^127^ and a primary invasive strategy is associated with a better prognosis.^124^^,^^127^ For non–ST-segment elevation MI (NSTEMI), a routine invasive strategy in all cancer patients is of unclear benefit.^124^^,^^128^ A recent retrospective study reported better survival with an invasive approach than with medical therapy in patients with NSTEMI and active cancer, including 12.9% with lung cancer.^128^ However, previous data in patients with metastatic disease, including metastatic lung cancer, suggest no benefit for invasive management.^124^^,^^129^ Although no randomized data exist for lung cancer, recent randomized controlled data suggest no benefit in the primary outcome (composite of cardiovascular death or nonfatal MI) routine invasive management in older patients presenting with NSTEMI^130^ and may apply to some patients with lung cancer. A tailored approach is therefore recommended, with consideration for shortening dual antiplatelet therapy and prioritizing radial access.^124^

#### Atrial fibrillation

Patients with active lung cancer and survivors are at high risk of developing AF.^119^^,^^131^ Nearly 20% of patients presenting to an outpatient lung cancer clinic have concurrent AF,^131^ and patients with lung cancer have an HR of 2.39 (95% CI: 2.30-2.48) of developing AF compared with non-cancer control subjects.^132^ There are conflicting data on whether the cancer itself is an independent risk factor for stroke in patients with AF. In a recent large population-based cohort study comparing the risk of stroke and bleeding in patients with AF and underlying malignancy vs cancer-free patients, the presence of cancer was not associated with a higher hazard of stroke but was associated with an increased hazard of bleeding (HR: 1.45; 95% CI: 1.37-1.53).^133^ However, the subgroup of patients with lung cancer (n = 4,234) was found to be at higher risk of both stroke (HR: 1.45) and bleeding (HR: 1.72) compared with matched control subjects without cancer after adjusting for anticoagulation.

Decisions regarding rate and rhythm management and anticoagulation are outlined in the recent European Society of Cardiology cardio-oncology guidelines.^84^ Recent data, which included a few patients with lung cancer (2 of 55), suggest that in patients with high risk of bleeding, left atrial appendage closure has similar outcomes in patients with cancer as those without at a median follow-up of 1.9 years; however, only 21.8% of patients (12 of 50) had active cancer.^134^ In patients who have hemodynamic instability or symptomatic AF, a rhythm control strategy may be required. In situations in which antiarrhythmic drug therapy is needed, careful review of drug interactions and risk assessment for worsening QTc prolongation are recommended, especially in patients being treated with targeted cancer therapy.^135^

#### Aortic stenosis

Patients with cancer commonly have AS due to shared risk factors, improved life expectancy, and use of radiation therapy.^136^ In patients with active cancer, transcatheter valve replacement (TAVR) is associated with reduced odds of acute kidney injury, cardiogenic shock, and bleeding with no difference in in-hospital mortality and stroke compared with surgical aortic valve replacement.^136^ Also, patients with active lung cancer have similar peri-procedural complications as patients without cancer when treated with TAVR.^137^ Patients with active lung cancer and severe AS require a multidisciplinary approach to treatment and should not be excluded from AS therapy based on cancer status alone.

### Summary

• Re-initiation of therapy after osimertinib or ICI cardiotoxicity requires a team-based approach and depends on the severity of the cardiotoxicity and the availability of alternative effective treatment.
• Patients with lung cancer and acute STEMI should be managed with a primary invasive strategy; for those with NSTEMI, a tailored approach to percutaneous intervention is recommended depending on stage of disease and expected survival.
• In patients with active lung cancer, TAVR is generally favored over surgical aortic valve replacement and has similar peri-procedural complications as patients without cancer.

## Conclusions

Although lung cancer remains the leading cause of cancer death worldwide, there has been significant improvement in cancer survival in recent years. Patients with lung cancer are at particularly high risk of prevalent and incident CVD, with an increasing impact on overall survival as prognosis of lung cancer improves. Although formal studies are required, optimization of CVD and CVD risk factors before treatment may improve access to broader treatment options and potentially lead to better treatment outcomes. Further data are needed to better guide surveillance strategies during cancer therapy, as well as the prevention and treatment of cardiotoxicity in patients receiving systemic therapy and radiation therapy for lung cancer.

## Funding Support and Author Disclosures

Dr Thavendiranathan is supported by a Tier II Canada Research Chair in Cardio-Oncology (#950-232646) and the Canadian Cancer Society/Canadian Institutes of Health Research’s W. David Hargraft Grant. Dr Yu is supported by funding from the Canadian Institutes of Health Research funding (#187923). The authors have reported that they have no relationships relevant to the contents of this paper to disclose.
